# Regularized Weighted Nonparametric Likelihood Approach for High-Dimension Sparse Subdistribution Hazards Model for Competing Risk Data

**DOI:** 10.1155/2021/5169052

**Published:** 2021-09-19

**Authors:** Leili Tapak, Michael R. Kosorok, Majid Sadeghifar, Omid Hamidi, Saeid Afshar, Hassan Doosti

**Affiliations:** ^1^Department of Biostatistics, School of Public Health, Hamadan University of Medical Sciences, Hamadan, Iran; ^2^Modeling of Noncommunicable Diseases Research Center, Hamadan University of Medical Sciences, Hamadan, Iran; ^3^Department of Biostatistics, Department of Statistics and Operations Research, University of North Carolina at Chapel Hill, USA; ^4^Department of Statistics, Bu-Ali Sina University, Hamedan, Iran; ^5^Department of Science, Hamedan University of Medical Science, Hamedan 65155, Iran; ^6^Research Center for Molecular Medicine, Hamadan University of Medical Sciences, Hamadan, Iran; ^7^Department of Mathematics and Statistics, Macquarie University, Sydney, Australia

## Abstract

Variable selection and penalized regression models in high-dimension settings have become an increasingly important topic in many disciplines. For instance, omics data are generated in biomedical researches that may be associated with survival of patients and suggest insights into disease dynamics to identify patients with worse prognosis and to improve the therapy. Analysis of high-dimensional time-to-event data in the presence of competing risks requires special modeling techniques. So far, some attempts have been made to variable selection in low- and high-dimension competing risk setting using partial likelihood-based procedures. In this paper, a weighted likelihood-based penalized approach is extended for direct variable selection under the subdistribution hazards model for high-dimensional competing risk data. The proposed method which considers a larger class of semiparametric regression models for the subdistribution allows for taking into account time-varying effects and is of particular importance, because the proportional hazards assumption may not be valid in general, especially in the high-dimension setting. Also, this model relaxes from the constraint of the ability to simultaneously model multiple cumulative incidence functions using the Fine and Gray approach. The performance/effectiveness of several penalties including minimax concave penalty (MCP); adaptive LASSO and smoothly clipped absolute deviation (SCAD) as well as their L_2_ counterparts were investigated through simulation studies in terms of sensitivity/specificity. The results revealed that sensitivity of all penalties were comparable, but the MCP and MCP-L_2_ penalties outperformed the other methods in term of selecting less noninformative variables. The practical use of the model was investigated through the analysis of genomic competing risk data obtained from patients with bladder cancer and six genes of CDC20, NCF2, SMARCAD1, RTN4, ETFDH, and SON were identified using all the methods and were significantly correlated with the subdistribution.

## 1. Introduction

The recent development of high-throughput biology provides powerful information about various phenotypic data including patients' survival times. One important task is to select a small subset of genes that are most relevant to survival outcomes [1, 2]. By uncovering the relationship between time to an event such as cancer and the expression profiles, one hopes to achieve more accurate prognoses and improved treatment strategies [3]. This issue is challenging for two main reasons. First, the number of covariates in microarray gene expression analysis or DNA sequencing data obtained from next-generation sequencing technology commonly far exceeds sample size (*p* > >*n*). Second, the availability and feasibility of standard analyses are severely affected by the high possibility of potential collinearity among different gene levels [2].

Variable selection techniques are powerful tools for sparse modeling in high-dimensional regression problems and finding the transcripts that most associate with the survival outcome, which aim to improve the predictive power and interpretability of the resulting model [4]. They are well developed in linear regression settings, and in recent years, many of them have been extended to the context of censored survival data [5]. For example, Cox-based methods utilizing the LASSO penalization [6–9], the elastic net (ENET) [1, 10], the nonconcave penalized likelihood approach [11], and smoothly clipped absolute deviation (SCAD) [12] have been proposed.

When data on patient survival time contains competing events, such as ‘progression' versus ‘death from noncancer cause,' often the standard analysis involves modeling the cause-specific hazards functions of the different failure types [13–15]. Nevertheless, “while the cause specific hazards is useful for investigating the disease dynamics to get insights in disease mechanisms and biological processes, it is less appropriate for clinical decision support for which it is preferable to consider the cumulative incidence probability, the marginal probability of failure for a specific cause” [16]. Moreover, the effect of a gene signature on the cause-specific hazards function of a particular failure type may be very different from its effect on the corresponding cumulative incidence function [13, 17, 18]. The synthesis interpretation of two cause-specific model fits is even more difficult in a high-dimensional setting, as the list of selected genes obtained from high-dimensional models are usually rather unstable [19]. So, under the cause-specific hazards formulation, it is not plausible to test the gene effects on the subdistribution, and the issues of model selection and efficient prediction cannot be directly addressed [13]. Some approaches have been proposed to deal with this situation. Fine and Gray [13] proposed a methodological framework for a formal direct synthesis model, which is the hazards attached to the cumulative incidence function. Their model adapts the semiparametric Cox proportional hazards model for the subdistribution hazard. “The method accommodates time-varying covariate effects on the subdistribution hazards and yields the usual nonparametric estimators in the absence of **z**” [20]. As the subdistribution hazards relates directly to the cumulative incidence function, only one model has to be fitted for describing the cumulative incidence function of the event of interest [19]. The estimation procedure in the proportional subdistribution hazards regression proposed by Fine and Gray [13] is based on a weighted partial likelihood function. Scheike et al. [21] introduced another approach to predict and model cumulative incidence probability by the direct binomial regression technique. They showed that this model is comparable with the Fine and Gray approach and can be fitted by standard packages. Other approaches include Andersen and Klein [22], Klein and Andersen [23], Fine [24], and Gerds et al. [25]. “None of the above methods adapt easily to time-varying covariates, which are most naturally accommodated in models for the hazards function, as with survival data without competing risks. Moreover, these methods do not reduce to the usual nonparametric estimators without covariates” [20].

Recently, some efforts have been made related to variable selection and direct modeling of cumulative incidence function for high-dimensional competing risk data including [19], Ambrogi and Scheike [16], Hou et al. [26], Hou et al. [27], Tapak et al. [28], Tapak et al. [29], Saadati et al. [30], Gilhodes et al. [31], and Fu et al. [32] based on different settings. None of the above approaches was likelihood-based procedures. In this regard, Bellach et al. [20] introduced “a weighted likelihood function that allows for a direct extension of the Fine and Gray model to a broad class of semiparametric regression models.” Considering this larger class of semiparametric regression models for the subdistribution is of particular importance, because the proportional hazards assumption may not be valid in general [20], especially in the high-dimension setting. Also, by considering this class of semiparametric regression models, the constraint of the ability to simultaneously model multiple cumulative incidence functions using the Fine and Gray approach is relaxed [20]. This model allows for time-dependent covariate effects on the subdistribution hazards as well [20]. Moreover, likelihood-based inference is permitted [20]. On the other hand, the available packages include “crrp” and “glmnet.” The current version 1.0 of “crrp” is designed for low-dimensional competing risk data and the “glmnet” provides only LASSO and elastic net penalties, and it is not possible to use other sparse penalties like the SCAD and the minimax concave penalty (MCP). Recently, Kawaguchi et al. [33] provided a R package named “fastcmprsk” for penalized variable selection with MCP, SCAD, LASSO, and elastic net penalties for competing risks based on the Fine and Gray model using subdistribution hazards model. They studied the performance of their model with *p* = 100 covariates and *n* = 1000 to 4000 sample sizes. The aim of the present study is to propose a penalized weighted nonparametric likelihood approach to regularized-based variable selection for competing risk data with high-dimensional covariates. This is the extension of the [20] to the high-dimension setting. We consider three popular penalties for individual variable selection: adaptive LASSO (ALASSO), SCAD, and minimax concave penalty (MCP). We also propose a group variable selection via elastic net (ENET), SCAD-L_2_, and MCP-L_2_. The proposed method, including the model, penalized likelihood approach, and estimation procedure, are described in Section 2. In Section 3, simulation studies are presented. An illustrative example using bladder cancer data is provided in Section 4. Some discussions are provided in Section 5.

## 2. Proposed Method

### 2.1. General Subdistribution Hazards Model

Following notations used by Fine and Gray [13], let T_*i*_ and *C*_*i*_ denote the failure time and the censoring time of the *i*th individual, respectively, with *X*_*i*_ = *T*_*i*_∧*C*_*i*_ as the observed time, and Δ_*i*_ = *I*{*T*_*i*_ ≤ *C*_*i*_∧*τ*} is the noncensoring indicator (*τ* is the maximum time of the study). Furthermore, *ε*_*i*_ ∈ {1, ⋯, *K*} specifies the potential type of the failure, and Z_*i*_(t) is a *d* × 1 possibly time-dependent covariate vector of bounded variation [13].

The focus of the present study is on modeling the cumulative incidence function for failure from cause 1, *F*_1_(*t*; **z**) = *P*(*T* ≤ *t*, *ε* = 1 | **z**). To estimate *F*_1_, the modeling of the subdistribution hazards for the event of interest was proposed by Fine and Gray [13] which leads to direct estimating of *F*_1_ without simultaneous estimating subdistribution functions corresponding to other failure types [34]. Specifically, the subdistribution hazards of the first event type are defined as follows:

(1) $$\begin{array}{c} \alpha_{1}\left(t;z\right)=\underset{\Delta t⟶0}{\lim}\frac{1}{\Delta t}P\left(\text{t}<T\leq t+\Delta t,\varepsilon =1\left|T\right.\geq t\cup \left(T\leq t\cap \varepsilon \neq 1\right),z\right). \end{array}$$

Considering *A*(*t*) as the cumulative subdistribution hazard, Bellach et al. [20] proposed the following general model for it:

(2) $$\begin{array}{c} A\left(t\right)=g\left(\int_{0}^{t}e^{\beta^{T}Z\left(s\right)}{dA}_{0}\left(s\right)\right), \end{array}$$

where *β* ∈ *R*^*d*^ stands for the regression parameters and *A*_0_ stands for an unspecified increasing function. Also, *g* is a thrice differentiable function which is strictly increasing and continuous with *g*(0) = 0, *g*′(0) > 0, and *g*(∞) = ∞. For other regularity conditions, see [20]. These conditions guarantee the existence of the weighted nonparametric maximum likelihood estimations. The *g*(.) can have different forms including *g*(*x*) = {(1 + *x*)^*ρ*^ − 1}/*ρ* for *ρ* ≥ 0 (the class of Box-Cox transformation models) and *g*(*x*) = log(1 + *rx*)/r for *r* ≥ 0 (the class of logarithmic transformation models) [20]. Both links result in the Fine and Gray model as a special case (let *ρ* = 1 in the first one and *r*⟶0 in the second link function).

### 2.2. Penalized Weighted Nonparametric Maximum Likelihood Estimation

Assume that there are no tied event times. With *N*(*t*) = ∑_*i*=1_^*n*^*N*_*i*_(*t*) (where*N*_*i*_(*t*) = *I*{*T*_*i*_ ≤ *t*, *ε*_*i*_ = 1}) as the counting process of the event of interest and *Y*(*t*) = ∑_*i*=1_^*n*^*Y*_*i*_(*t*) as the at risk indicator, the weighted log-likelihood function under the general semiparametric regression model is as follows:

(3) $$\begin{array}{c} l\left(\beta ,A_{0}\right)=\overset{n}{\underset{i=1}{\sum }}\left[\int_{0}^{\tau }\log\left(e^{\beta^{T}Z_{i}\left(t\right)}\alpha_{0}\left(t\right)g^{\prime }\left(\int_{0}^{t}e^{\beta^{T}Z_{i}\left(u\right)}{dA}_{0}\left(u\right)\right)\right)I\left(C_{i}\geq t\right)Y_{i}\left(t\right){dN}_{i}\left(t\right)-\int_{0}^{\tau }w_{i}\left(t\right)Y_{i}\left(t\right)e^{\beta^{T}Z_{i}\left(t\right)}g^{\prime }\left(\int_{0}^{t}e^{\beta^{T}Z_{i}\left(u\right)}{dA}_{0}\left(u\right)\right){dA}_{0}\left(t\right)\right], \end{array}$$

where *w*_*i*_(*t*) is obtained by using inverse probability of censoring weighting (IPCW) technique with $w_{i}\left(t\right)=\text{I}\left\{C_{i}\geq T_{i}\wedge t\right\}.{\hat{G}}_{C}\left(t\right)/{\hat{G}}_{C}\left(T_{i}\wedge t\right)$ (where ${\hat{G}}_{C}$ is the product limit estimator of *G*_*C*_(*t*) = P(C > *t*)).

We now define the regularized estimator $\hat{\beta }$ as a solution to the regularization problem:

(4) $$\begin{array}{c} \hat{\beta }=\underset{\beta \in R^{d}}{\arg\max}\left\{l_{\text{pen}}\left(\beta ,A_{0}\right)=l\left(\beta ,A_{0}\right)+\overset{d}{\underset{j=1}{\sum }}p_{\lambda }\left(\left|\beta_{j}\right|\right)\right\}, \end{array}$$

where *p*_*λ*_(.) is a penalty function that depends on the regularization parameter *λ* ≥ 0. The cumulative baseline hazards *A*_0_ is approximated by a sequence of step functions (*A*_*n*_^0^) with jumps at the observed events of interest. By considering the $0<{\tilde{T}}_{j}<\tau ;0<j<k\left(n\right)$ as the ordered times with *k*(*n*) be the number of the events of interest and replacing *A*_0_ by *A*_*n*_^0^, a modified penalized likelihood function, *l*_pen,*n*_(*β*, *A*_*n*_^0^), is obtained which is maximized to yield the regularized estimators of the regression coefficients. In the maximization process, the estimators of *A*_*n*_^0^ are obtained as $A_{n}^{0}\left\{{\tilde{T}}_{j}\right\}$, where $A_{n}^{0}\left\{{\tilde{T}}_{j}\right\}=A_{n}^{0}\left({\tilde{T}}_{j}\right)-A_{n}^{0}\left({\tilde{T}}_{j-1}\right)$ [20].

In the absence of covariates, a Nelson-Aalen type estimator of the subdistribution hazards is obtained by using the weighted likelihood function [20] which is derived from the weighted Doob decomposition *w*_*i*_(*t*)*dN*_*i*_(*t*) = *w*_*i*_(*t*)*Y*_*i*_(*t*)*α*(*t*)*dt* + *w*_*i*_(*t*)*dM*_*i*_(*t*), with

(5) $$\begin{array}{c} \overset{n}{\underset{i=1}{\sum }}w_{i}\left(t\right)Y_{i}\left(\text{t}\right)=\overset{n}{\underset{i=1}{\sum }}I\left\{X_{i}\geq t\right\}+\overset{n}{\underset{i=1}{\sum }}I\left\{X_{i}<t,\Delta_{i}=1,\varepsilon_{i}\neq 1\right\}\frac{{\hat{G}}_{C}\left(t\right)}{{\hat{G}}_{C}\left(T_{i}\wedge t\right)}, \end{array}$$

which is the expected #subjects in the pseudorisk set [20]. Also, by considering the jump sizes of the baseline as a parameter, maximization of the following discretized log-likelihood:

(6) $$\begin{array}{c} l=\overset{n}{\underset{i:\Delta_{i}\varepsilon_{i}=1}{\sum }}\left\{\log A_{n}\left\{X_{i}\right\}+\beta^{T}Z_{i}\left(X_{i}\right)+\log\left(g^{\prime }\left(\underset{\begin{array}{c} j:X_{j}\leq X_{i}\\ \Delta_{j}\varepsilon_{j}=1 \end{array}}{\sum }e^{\beta^{T}Z_{i}\left(X_{j}\right)}A_{n}\left\{X_{j}\right\}\right)\right)\right\}-\overset{n}{\underset{i=1}{\sum }}g\left(\underset{k:\Delta_{k}\varepsilon_{k}=1}{\sum }I\left(\left(X_{i}\wedge \tau \right)\geq X_{k}\right)e^{\beta^{T}Z_{i}\left(X_{k}\right)}A_{n}\left\{X_{k}\right\}\right)\underset{i:\Delta_{i}\varepsilon_{i}=2}{\sum }\left(\underset{k:\Delta_{k}\varepsilon_{k}=1}{\sum }w_{i}^{*}\left(X_{k}\right)I\left(X_{i}\geq X_{k}\right)e^{\beta^{T}Z_{i}\left(X_{k}\right)}A_{n}\left\{X_{k}\right\}g^{\prime }\left(\underset{\begin{array}{c} j:X_{j}\leq X_{k}\\ \Delta_{j}\varepsilon_{j}=1 \end{array}}{\sum }e^{\beta^{T}Z_{i}\left(X_{j}\right)}A_{n}\left\{X_{j}\right\}\right)\right), \end{array}$$

will yield the estimator of the parameters.

In this study, we only considered *g*(*x*) = *x* and *g*(*x*) = log(1 + *x*) which corresponds to the proportional subdistribution hazards model and proportional odds model (nevertheless, the method can be extended to other link functions). Then, the weighted log-likelihood function takes the following form:

(7) $$\begin{array}{c} l\left(\beta ,A_{0}\right)=\overset{n}{\underset{i=1}{\sum }}\left[\int_{0}^{\tau }\log\left(e^{\beta^{T}Z_{i}\left(t\right)}\alpha_{0}\left(t\right)\right)I\left(C_{i}\geq t\right)Y_{i}\left(t\right){dN}_{i}\left(t\right)-\int_{0}^{\tau }w_{i}\left(t\right)Y_{i}\left(t\right)e^{\beta^{T}Z_{i}\left(t\right)}{dA}_{0}\left(t\right)\right]. \end{array}$$

This can be factorized into two parts including the Fine and Gray partial likelihood function and a second term:

(8) $$\begin{array}{c} L_{n}=\underset{i:\Delta_{i}\varepsilon_{i}=1}{\prod }\left[\frac{w_{i}\left(X_{i}\right)Y_{i}\left(X_{i}\right)e^{\beta^{T}Z_{i}\left(X_{i}\right)}}{\sum_{j=1}^{n}w_{i}\left(X_{i}\right)Y_{i}\left(X_{i}\right)e^{\beta^{T}Z_{i}\left(X_{i}\right)}}\right].\left(\overset{n}{\underset{j=1}{\sum }}w_{i}\left(X_{i}\right)Y_{i}\left(X_{i}\right)e^{\beta^{T}Z_{i}\left(X_{i}\right)}A_{0}\left\{X_{i}\right\}\right)\times \exp\left(-\int_{0}^{\tau }w_{i}\left(t\right)Y_{i}\left(t\right)e^{\beta^{T}Z_{i}\left(t\right)}{dA}_{0}\left(u\right)\right). \end{array}$$

Without penalty term, for *g*(*x*) = *x*, estimation of parameters derived from the weighted log-likelihood function is identical to the estimations derived from the Fine and Gray model [20].

In this study, we considered the following penalties:

1. The adaptive LASSO (Zou 2006): *p*_*λ*_(|*β*_*j*_|) = *λυ*_*j*_|*β*_*j*_| ($\upsilon_{j}=1/\left|{\hat{\beta }}_{j}\right|$ is a data-driven weight)
2. The SCAD [11]: *p*_*λ*_′(|*β*_*j*_|) = *λI*(|*β*_*j*_| ≤ *λ*) + ((*αλ* − |*β*_*j*_|)_+_/(*α* − 1))*I*(|*β*_*j*_| > *λ*), where *α* > 2 is a tuning parameter
3. The MCP [35], *p*_*λ*_′(|*β*_*j*_|) = (*λ* − |*β*_*j*_|/*γ*)_+_, where *γ* > 1 is a tuning parameter
4. The adaptive elastic net (AENET) (Zou 2006): *p*_*λ*_(|*β*_*j*_|) = *λ*_1_*υ*_*j*_|*β*_*j*_| + *λ*_2_|*β*_*j*_|^2^ ($\upsilon_{j}=1/\left|{\hat{\beta }}_{j}\right|$ is a data-driven weight).
5. The SCAD-L_2_ Zeng and Xie 2020 [36] and MCP-L_2_ penalties, where a L_2_ penalty is appended to the SCAD and MCP penalties to induce grouping effect in variable selection

Asymptotic properties of penalized estimators in different contexts have been investigated by different studies, and all the above penalties have been shown to enjoy the oracle property [26, 27, 32], i.e., these penalties are consistent in variable selection, and their estimators are asymptotically normal and unbiased. More explicitly, they work as well as knowing the true model in advance. Fan and Li [11] established the oracle property and the asymptotic normality of a general class of nonconcave penalized maximum likelihood estimators with diverging number of parameters and increasing sample size and provided conditions to establish oracle property. In the framework of the subdistribution hazards model, Fu et al. [32] showed that ALASSO, SCAD, and MCP penalized estimators obtained from the Fine and Gray model (as a special case of model (2)) possess the oracle properties and the asymptotic normality. They established a theorem that if a penalty term (say, *p*_*λ*_*n*__(|*β*|)) simultaneously satisfies the two following conditions for *a*_*n*_ = max{*p*_*λ*_*n*__′(|*β*_*j*0_|): *β*_*j*0_ ≠ 0} and *b*_*n*_ = max{*p*_*λ*_*n*__^″^(|*β*_*j*0_|): *β*_*j*0_ ≠ 0}: (1) *a*_*n*_ = *O*_*p*_(*n*^−1/2^) and *b*_*n*_⟶0 and (2) for any *C* > 0, $\underset{n⟶\infty }{\lim}\sqrt{n}\underset{\left|\beta \right|\leq {Cn}^{-1/2}}{\inf}p_{\lambda_{n}}^{\prime }\left(\left|\beta \right|\right)⟶\infty$; then, the estimator enjoys the consistency in variable selection and asymptotical normality and unbiasedness. As Bellach et al. [20] showed that the Fine and Gray model is a special case of model provided in equation (2) (weighted NPMLE method), so the same results hold here under regularity conditions for the weighted likelihood.

### 2.3. Computational Algorithm

To compute the coefficients, several algorithms have been suggested by different authors to optimize equation (3), including the path algorithm [7] and LARS [37]. However, the maximization in this paper was utilized through the efficient algorithm proposed by Goeman [38], which is a combination of gradient ascent optimization with the Newton-Raphson algorithm. This algorithm, a full gradient algorithm, follows the gradient of the likelihood from a given starting value of *β*. But, unlike the coordinatewise gradient approach, it uses the full gradient at each step instead of updating a single coordinate at a time. Moreover, the algorithm automatically switches to a Newton-Raphson algorithm when it gets close to the optimum to avoid slow convergence.

The weighted log-likelihood function in equation (3) and the *ℓ_2_* penalty term in equation (4) are highly regular functions in terms of being concave and at least twice differentiable everywhere. The L_1_ penalty is less well-behaved as it is concave and continuous but is only differentiable at points with *β*_*i*_ ≠ 0 for all *i*. Therefore, the conditions needed to apply the gradient ascent algorithm with Newton-Raphson steps need to be verified. Let us consider $l_{\text{pen}}^{\prime }\left(\beta ;\nu \right)=\underset{t↓0}{\lim}\left(1/t\right)\left\{l_{\text{pen}}\left(\beta +t\nu \right)-l_{\text{pen}}\left(\beta \right)\right\}$ and $l_{\text{pen}}^{\prime \prime }\left(\beta ;\nu \right)=\underset{t↓0}{\lim}\left(1/t\right)\left\{l_{\text{pen}}^{\prime }\left(\beta +t\nu \right)-l_{\text{pen}}^{\prime }\left(\beta \right)\right\}$ be the directional derivative and directional second derivative of the penalized likelihood defined in equation (3) for every *β* in every direction *ν* ∈ *R*^*d*^, respectively. Then, the gradient can be defined for any *β* as the scaled direction of the steepest ascent. Also, let *ν*_opt_ (opt stands for optimum) be the direction that maximizes *l*_pen_′(*β*; *ν*) among all *v* with ‖*v*‖ = 1, *l*_pen_′ is the derivative of penalized log-likelihood.

We utilized the following algorithm, proposed by Goeman [38], to compute coefficients:

1. Start with some *β*_0_ (e.g., obtained from fitting the univariate Fine and gray model) and *A*_0_(*t*) = *n*^−1^
2. For steps *m* = 0, 1, 2, ⋯, iterate

(9) $$\begin{array}{c} {\hat{A}}_{0}^{\left(m+1\right)}\left\{X_{j}\right\}=\frac{1}{n}{\left[.\Phi_{n}\left({\text{T}}_{i},{\overset{\wedge }{A}}_{n}^{0},{\overset{\wedge }{\beta }}^{\left(m\right)}\right)\right]}^{-1}, \end{array}$$

where

(10) $$\begin{array}{c} \Phi_{n}\left(X_{j},{\hat{A}}_{n}^{0},{\overset{\wedge }{\beta }}^{\left(m\right)}\right)=\frac{1}{n}\overset{n}{\underset{k=1}{\sum }}I\left(\left(X_{k}\wedge \tau \right)\geq X_{j}\right)e^{{\overset{\wedge }{\beta }}^{\left(m\right)T}Z_{k}\left(X_{j}\right)}g^{\prime }\left\{\int_{0}^{X_{k}}e^{{\overset{\wedge }{\beta }}^{\left(m\right)T}Z_{k}\left(u\right)}d{\hat{A}}_{n}^{0}\left(u\right)\right\}-\frac{1}{n}\overset{n}{\underset{k=1}{\sum }}I\left(X_{k}\geq X_{j}\right)e^{{\overset{\wedge }{\beta }}^{\left(m\right)T}Z_{k}\left(X_{j}\right)}\int_{X_{j}}^{\tau }\frac{g^{\prime \prime }\left\{\int_{0}^{X_{k}}e^{{\overset{\wedge }{\beta }}^{\left(m\right)T}Z_{k}\left(u\right)}d{\hat{A}}_{n}^{0}\left(u\right)\right\}}{g^{\prime }\left\{\int_{0}^{X_{k}}e^{{\overset{\wedge }{\beta }}^{\left(m\right)T}Z_{k}\left(u\right)}d{\hat{A}}_{n}^{0}\left(u\right)\right\}}I\left(C_{k}\geq t\right){dN}_{k}\left(t\right)+\frac{1}{n}\overset{n}{\underset{k:\Delta_{k}\varepsilon_{k}=1}{\sum }}I\left(X_{k}\leq X_{j}\right)w_{k}^{*}\left(X_{j}\right)e^{{\overset{\wedge }{\beta }}^{\left(m\right)T}Z_{k}\left(X_{j}\right)}g^{\prime }\left\{\int_{0}^{X_{j}}e^{{\overset{\wedge }{\beta }}^{\left(m\right)T}Z_{k}\left(u\right)}d{\hat{A}}_{n}^{0}\left(u\right)\right\}+\frac{1}{n}\overset{n}{\underset{k:\Delta_{k}\varepsilon_{k}=1}{\sum }}I\left(X_{k}\leq X_{j}\right)e^{{\overset{\wedge }{\beta }}^{\left(m\right)T}Z_{k}\left(X_{j}\right)}\int_{X_{k}}^{\tau }w_{k}^{*}\left(t\right)e^{{\overset{\wedge }{\beta }}^{\left(m\right)T}Z_{k}\left(t\right)}g^{\prime \prime }\left\{\int_{0}^{t}e^{{\overset{\wedge }{\beta }}^{\left(m\right)T}Z_{k}\left(u\right)}d{\hat{A}}_{n}^{0}\left(u\right)\right\}d{\hat{A}}_{n}^{0}\left(t\right),\\ \beta^{\left(m+1\right)}=\left\{\begin{array}{c} \beta^{\left(m\right)}+t_{\text{edge}}\kappa \left(\beta^{\left(m\right)}\right),\\ \beta_{\text{NR}}^{\left(m\right)},\\ \beta^{\left(m\right)}+t_{\text{opt}}\kappa \left(\beta^{\left(m\right)}\right), \end{array}\begin{array}{c} \text{if }t_{\text{opt}}\geq t_{\text{edge}},\\ \text{if }t_{\text{opt}}\leq t_{\text{edge}}\text{ and }\mathrm{sign}\left(\beta_{\text{NR}}^{\left(m+1\right)}\right)=-\mathrm{sign}\left(\beta_{+}^{\left(m\right)}\right),\\ \text{o}.\text{w}, \end{array}\right. \end{array}$$

until convergence.

In the above algorithm *κ*(*β*) = (*κ*_1_(*β*), ⋯,*κ*_*d*_(*β*))′ is the gradient vector and is calculated as

(11) $$\begin{array}{c} \kappa \left(\beta^{\left(m\right)}\right)=\left\{\begin{array}{cc} l_{\text{pen}}^{\prime }\left(\beta ;v_{\text{opt}}\right)\cdot v_{\text{opt}}, & \text{if }l_{\text{pen}}^{\prime }\left(\beta ;v_{\text{opt}}\right)\geq 0,\\ 0, & \text{otherwise}, \end{array}\right. \end{array}$$

and $t_{\text{edge}}=\underset{i}{\min}\left\{-\left(\beta_{i}/\kappa_{i}\left(\beta \right)\right)\text{: }\mathrm{sign}\left(\beta_{i}\right)=-\mathrm{sign}\left\{\kappa_{i}\left(\beta \right)\right\}\neq 0\right\}$, *t*_opt_ = −(*l*_pen_′(*β*; *κ*(*β*))/*l*_pen_^″^(*β*; *κ*(*β*))), *β*_*NR*_ is the Newton-Raphson estimator and sign(*β*_+_) = lim_*ψ*↓0_sign(*β* + *ψκ*(*β*)).

For the general form shown in equation (3),

(12) $$\begin{array}{c} \frac{\partial l_{\text{pen}}\left(\beta ,A_{0}\right)}{\partial \beta }=\overset{n}{\underset{i=1}{\sum }}\left[\int_{0}^{\tau }Z_{i}\left(t\right)g^{\prime }\left(\int_{0}^{t}e^{\beta^{T}Z_{i}\left(u\right)}{dA}_{0}\left(u\right)\right)+\beta^{T}Z_{i}\left(t\right)g^{\prime \prime }\left(\int_{0}^{t}e^{\beta^{T}Z_{i}\left(u\right)}{dA}_{0}\left(u\right)\right)I\left(C_{i}\geq t\right)Y_{i}\left(t\right){dN}_{i}\left(t\right)\right.-\int_{0}^{\tau }\frac{w_{i}\left(t\right)Y_{i}\left(t\right)Z_{i}\left(t\right)e^{\beta^{T}Z_{i}\left(t\right)}g^{\prime }\left(\int_{0}^{t}e^{\beta^{T}Z_{i}\left(u\right)}{dA}_{0}\left(u\right)\right)+w_{i}\left(t\right)Y_{i}\left(t\right)e^{\beta^{T}Z_{i}\left(t\right)}g^{\prime \prime }\left(\int_{0}^{t}e^{\beta^{T}Z_{i}\left(u\right)}{dA}_{0}\left(u\right)\right)}{w_{i}\left(t\right)Y_{i}\left(t\right)e^{\beta^{T}Z_{i}\left(t\right)}g^{\prime }\left(\int_{0}^{t}e^{\beta^{T}Z_{i}\left(u\right)}{dA}_{0}\left(u\right)\right)}{dA}_{0}\left(t\right)+p_{\lambda_{n}}^{\prime }\left(\left|\beta \right|\right). \end{array}$$

### 2.4. Tuning Parameters

There are various ways to find optimal penalized estimators including cross-validation (CV; which requires randomly splitting the data), generalized cross-validation (GCV), and Bayesian information criterion (BIC). As the random nature of splitting the data in CV makes the tuning parameters unstable [39] and the GCV may lead to the overfitting effect on the resulting model [35], we used the BIC, which has been shown to be consistent in identifying the true model [39]:

(13) $$\begin{array}{c} \text{BIC}=-2l\left(\tilde{\beta },A_{0}\right)+\log\left(n\right)s\left(\lambda \right), \end{array}$$

where *l* is the weighted log-likelihood function, $\tilde{\beta }$ maximizes the weighted log-likelihood function (the penalized estimator), and *s*(*λ*) is the size of the model (the number of nonzero coefficients) [35].

## 3. Simulation Study

The proposed variable selection method was investigated through different simulation scenarios. Competing risk data with two possible events (causes of failure) were simulated, where the event of type 1 was the one of interest (I) and the event of type 2 was the competing risk (*C*). To construct a high-dimension setting, *d* = 5000 covariates were considered with *n* = {200, 400} observations. Among covariates, similar to other studies, 5 informative covariates with effects on the subdistribution hazards for events of type 1 and/or 2 were considered; the vector of regression parameters for cause 1 was considered $\beta_{1}=\left(\underset{5}{\underbrace{0.5,0.5,-.05,0.5,-0.5}},\underset{4995}{\underbrace{0,\cdots ,0}}\right)$, and for cause 2, it was *β*_2_ = −*β*_1_. The values of 0.5 and -0.5 were considered for increasing and decreasing effects, and for the covariates with no direct effect on the hazards, the value of 0 was considered. In all scenarios, covariates were generated from a multivariate normal distribution with mean zero and covariance matrix (*ρ*^|*i* − *j*|^)_*i*,*j*=1_^*d*^. We considered *ρ* = {0.1, 0.5} as in Lin and Lv [4].

Following the strategy used by Fine and Gray [13], event times were generated based on proportional subdistribution hazards. To this end, after generating covariates, considering *k* as the ratio of *I*/*C*, the subdistribution for the first event (type 1) was generated as follows:

(14) $$\begin{array}{c} \mathrm{Pr}\left(T_{i}\leq t,\varepsilon_{i}=1\left|z_{i}\right.\right)=1-{\left[1-k\left\{1-\exp\left(-t\right)\right\}\right]}^{\exp\left(\overset{d}{\underset{i=1}{\sum }}\beta_{i1}z_{i}\right)}, \end{array}$$

which is a unit exponential mixture, with mass 1 − *k* at ∞ when all covariates are zero. The subdistribution for the second event type was generated using an exponential distribution with rate exp(∑_*i*=1_^*d*^*β*_*i*1_**z**_*i*_) by taking Pr(*ε*_*i*_ = 2 | **z**_i_) = 1 − Pr(*ε*_*i*_ = 1 | **z**_i_). Moreover, we considered proportional odds model. So, in this setting, the subdistribution for the events was defined by *F*_1_(*t* | *Z*_*i*_) = exp[*k* + log{1 − exp(−1)} + ∑_*i*=1_^*d*^*β*_*i*1_**z**_*i*_](1 + exp[*k* + log{1 − exp(−1)} + ∑_*i*=1_^*d*^*β*_*i*1_**z**_*i*_])^−1^. Censoring times were generated from a uniform *U*(0, *a*) distribution. The value of *a* = 3 was selected to yield average censoring rate for 40% of the observations. We also considered *k* = {0.2, 0.5, 0.8}. Because the calculated estimations for the coefficients were biased toward zero, we focused on the accuracy of variable selection (relevant covariates). Therefore, variable selection was expressed in terms of the sensitivity or the true positive rate (TPR; the number of correctly identified informative/relevant variables (true positives; variables that associated with to the cumulative incidence function of the event of interest, say event (1) divided by the total number of informative variables) and the false positive rate (FPR; the number of unrelated variables chosen divided by the total number of irrelevant variables) with respect to the event of interest (e.g., event 1). For the sake of comparison, the boosted subdistribution hazards regression model [19] was considered and implemented in R package CoxBoost [40]. The constant *a* in the SCAD and SCAD-L_2_ penalty functions was fixed as *a* = 3.7.

The mean and standard deviation of different scenarios (twelve scenarios) over 500 replicates were summarized in Tables 1 and 2, respectively.

Table 1 shows the results for independent covariate scenarios. As seen, ALASSO and AENET had a very close performance in this setting and MCP and MCP-L_2_ outperformed ALASSO and AENET in that they selected sparser models with a better sensitivity and a greater specificity or a lower FPR (a better ability in eliminating irrelevant variables). Moreover, as expected due to the similarity, SCAD, SCAD-L_2_, MCP, and MCP-L_2_ had comparable performance, of which MCP and MCP-L_2_ selected a slightly sparser model than the SCAD and SCAD-L_2_. Generally, all penalized estimators get at least three out of the five relevant nonzero variables. For *k* = 0.2 and *n* = 200, the sensitivities are the lowest compared with other scenarios. Considering TPR, Boosting outperformed the penalized methods, especially in the settings *k* = 0.2. As the *k* increases from 0.2 to 0.8, the TPR of the penalized methods became closer to TPR of the Boosting method. On the other hand, considering FPR, Boosting showed similar performance with the penalized methods for the *k* = 0.2 setting. Nevertheless, as the *k* increases from 0.2 to 0.8, the FPR of the penalized methods decreases, while there was no change in the FPR of Boosting. Simulation studies of other studies [16] showed that the results of Boosting method is in line with the nonconcave penalty of LASSO which tends to include more irrelevant variables. Moreover, the “CoxBoost” package uses cross-validation method to choose tuning parameters (a prediction-based criterion), which predispose the method to include too many irrelevant variables in LASSO type procedures [41, 42]. Moreover, in general, for both *n* = 200 and *n* = 400 settings, it was observed that the performance the MCP and MCP-L_2_ performed best among the others, with a performance very close to that of the oracle estimator especially when the ratio of *I*/*C* is 0.8 (the lower rate of competing event). This finding was in concordance with the results of Lin and Lv [4] where they proposed penalized additive hazards models for survival data analysis with one failure cause.

Table 2 shows the results for moderate correlation between covariates (*ρ* = 0.5). Again, MCP and MCP-L_2_ outperformed other methods in almost all scenarios and its performance was closer to that of the oracle estimator, especially when *n* = 400 and *k* = 0.8. As there was moderate correlation between covariates, the AENET, SCAD-L_2_, and MCP-L_2_ penalties showed a greater TPR compared with the L_1_ penalties including ALASSO, SCAD, and MCP. For all methods, the sensitivities increase and FPRs decrease as *k* increases from 0.2 to 0.5 and 0.8. For *n* = 400, the average number of selected variables decreases slightly and better sensitivities were resulted in compared with *n* = 200. Comparing the results provided in Tables 1 and 2, it was revealed that in the presence of moderate correlation compared with low correlation, the TPRs diminish for all penalized models. However, the Boosting method is almost robust to moderate correlation.

We also conducted simulations with various regression coefficients. So, $\beta_{1}=\left(\underset{5}{\underbrace{0.2,-0.4,0.5,-0.8,0.6}},\underset{4995}{\underbrace{0,\cdots ,0}}\right)$ and the place of nonzero elements of *β*_2_ was selected randomly, while holding censoring rates and correlations constant. Results were shown in supplementary file (Table S1). According to the results, the selection results were robust. These results were in accordance with the findings in Fu et al. [32] and Zhang and Lu [43].

We also designed some scenarios for proportional odds method with *g*(*x*) = log(1 + *x*).  Table 3 shows the results of the simulation studies for proportional odds model (*g*(*x*) = log(1 + *x*)) with 5 informative variables for *ρ* = 0.1 and *ρ* = 0.5, about 40% average censoring and *k* = *I*/*C* = 0.5. According to the results, the performance of different penalties in the proportional odds model was similar to those of the proportional hazards and again the MCP and MCP-L_2_ outperformed the ALASSO and AENET in terms of greater TPR and lower FPR.

To investigate the grouping effect or the performance of the models in the presence of high correlations in high-dimensional settings, we also considered high correlations. In this regard, following the strategy considered by Zeng and Xie [36], two groups were considered for informative variables, each including 3 variables as follows:

(15) $$\begin{array}{c} \begin{array}{ccc} z_{i}=Z_{1}^{\prime }+e_{i}, & Z_{1}^{\prime }\sim N\left(0,1\right), & i=1,2,3,\\ z_{i}=Z_{2}^{\prime }+e_{i}, & Z_{2}^{\prime }\sim N\left(0,1\right), & i=4,5,6,\\ z_{i}\sim N\left(0,1\right) & i=7,\cdots ,5000 \end{array} \end{array}$$

with $e_{i}\overset{i.i.d.}{\sim }N\left(0,0.01\right)$, *i* = 1, ⋯, 6.

The results were reported in terms of selection accuracy illustrated by the number of nonzero coefficients correctly identified (TPR) and the number of zero coefficients misspecified as nonzero coefficients (FPR).  Table 4 illustrates variable selection accuracy of different methods. For noninformative variables, we summarized the results as the average of FPR over 500 repetitions. From Table 4, we see that, in all settings, the variables *X*_1_, *X*_2_, and *X*_5_ were selected by all methods. In the presence of high correlations, the rate of model misspecification was high, which was due to the fact that the MCP, SCAD, and ALASSO penalties and Boosting tend to select only one variable from a group of variables that are highly correlated and it is not important which one is selected. However, the three penalties of SCAD-L_2_, MCP-L_2_, and AENET selected *X*_3_, *X*_4_, and *X*_6_ in addition to *X*_1_, *X*_2_, and *X*_5_ in all settings indicating that they enjoy the grouping effect which has been discussed by Zou and Hastie [44] and Huang et al. [45]. These findings were in concordance with those of other studies with other responses [1].

## 4. Application to Bladder Cancer Data

We used a publicly available time-to-event dataset with competing risks which corresponds to preprocessed 1381 custom platform microarray features (GEO with series accession no.GSE5479) from patients with bladder cancer to illustrate the proposed techniques. Bladder cancer is a common malignant disease with two different forms including non–muscle-invasive tumors (stages Ta and T1) and muscle-invasive cancers (stages T2-T4) [46]. This dataset includes information about a sample of *n* = 404 patients with pT*a* and pT1 tumors, with no previous or synchronous muscle-invasive tumors. In addition to gene expression measurements, this dataset contains potentially important clinical covariates including age, sex, stage (pTa versus pT1), grade (PUNLMP/low versus high), and treatment. There was complete information for only *n* = 301 patients, and we limit our analysis to this subset. There were also two competing events: time to progression or death from bladder cancer (the event of interest) and death from other or unknown causes. Progression or death from bladder cancer and competing events were observed in 74 and 33 patients, respectively. In addition, there was censoring for 194 patients [46].

The proposed method was applied to this microarray bladder cancer data for ‘progression or death from bladder cancer' as the event of interest.  Table 5 shows gene signatures selected by each method. ALASSO, AENET, SCAD, SCAD-L2, MCP, MCP-L2, and Boosting selected 18, 19, 26, 22, 20, 21, and 12 genes, respectively. As can be seen, there are several genes that are related to bladder cancer biologically. Among all genes selected, there were six genes of CDC20, NCF2, SMARCAD1, RTN4, ETFDH, and SON selected by all methods.  Table 6 shows regression coefficients of six common genes selected by all methods correlated with bladder cancer patients' subdistribution hazards. According to the results, increasing the expression of CDC20 increases the incidence of death from bladder cancer (or progression of the disease) by 2.68 times. Moreover, increasing the expressions of NCF2, ETFDH, and SON genes are positively correlated with the incidence of death from bladder cancer. On the other hand, increasing the expression of SMARCAD1 and RTN4 decreases the incidence of death from bladder cancer.

Electron transfer flavoprotein dehydrogenase (ETFDH), a mitochondrial inner membrane protein, plays an essential role in the electron transfer chain [47]. The expression level of ETFDH correlates with overall survival in hepatocellular carcinoma patients [48]. Reticulon-4 (RTN4) has an essential role in cancer development and progression. The expression level of RTN4 was associated with patients' survival for several cancers [49, 50]. Neutrophil cytosolic factor 2 (NCF2), as a novel target of P53, has a critical role in cancer progression [51]. SON DNA-binding protein (SON) plays role in mRNA transcription and pre-mRNA splicing. Moreover, SON can control macrophage activities and cell cycle progression [52]. A recent study by Furukawa et al. indicated that SON has an essential role in pancreatic cancer proliferation and tumorigenesis [53]. SMARCAD1 has a critical role in chromatin remodeling and control gene expression. On the other hand, SMARCAD1 plays an essential role in the homologous recombination (HR) process for DNA double-strand break (DSB) repair. Recent studies show that SMARCAD1 involve in the proliferation and progression of pancreatic and breast cancers [54, 55]. CDC20 (Cell Division Cycle 20) encodes a regulatory protein that is an essential cell cycle regulator. Recent studies indicated that CDC20 dysregulation is correlated with tumor progression and prognosis in several cancers [56].

## 5. Discussion and Conclusions

Unique challenges are created in statistics due to rapid accumulation of massive information for patients in medical researches. In this regard, the need for selecting informative variables and eliminating noise variables (e.g., noninformative variables) as an important issue highlights the necessity for novel robust data analysis methods. This study proposed a penalized weighted nonparametric likelihood-based approach for sparse variable selection in high-dimension competing risk data setting. The proportional hazards model may not be satisfied for some covariates, and it cannot be assessed in high-dimension setting. The proposed model allows for taking into account time-varying effects. Also, this model relaxes the constraint of the ability to simultaneously model multiple cumulative incidence function using the Fine and Gray approach. As in nonpenalized setting [20], the regularized weighted nonparametric likelihood approach is extendable to a general class of semiparametric transformation models even to nonproportional subdistribution hazards setting.

We evaluated the performances of several penalties, including ALASSO, AENET, SCAD, and MCP, and their L_2_ counterparts called SCAD-L_2_ and MCP-L_2_ empirically through comprehensive simulations in high-dimensional settings with different covariate structures in terms of TPR and FPR. Although, the penalized proportional subdistribution hazards model have been proposed in previous studies, this study considered more scenarios with more penalties. Other works considered only low dimension with different penalties [32] or high dimension with a few L_1_ penalties with no more than 1000 variables [26, 27]. Our findings revealed that sensitivity of all penalties were comparable, but the MCP and MCP-L_2_ penalties outperformed the other methods in term of selecting less noninformative variables. Also, the results of MCP and MCP-L_2_ were closer to the oracle estimator compared with other penalties. For correlated structures, the penalties with L_2_ term including SCAD-L_2_, MCP-L_2_, and AENET enjoyed the grouping effect and showed better performance which was in concordance with similar studies with other responses like count [57]. Moreover, Fu et al. [32] established asymptotic properties of penalized estimators obtained from the Fine and Gray model. In the framework of the Cox model, Fan and Li [58] extended these properties to the Cox proportional hazards model [58] which is a special case of NPMLE (nonweighted). While there are special cases of the weighted NPMLE that the oracle property of the penalized estimators has been established, there is a need to investigate conditions for the general class of models in equation (2) theoretically, especially for the time-varying covariates framework. So, this would be a subject for future studies.

One useful feature of the penalized weighted nonparametric maximum likelihood approach is that the AIC and BIC can easily be calculated as a simple tool for model selection, as it was used here. This resulted in a more stable variable selection approach compared with the models that uses cross-validation.

Variable selection in the survival setting, in general, is a difficult issue and is even more challenging in the competing risk setting. As a result, a relatively large sample size is required to make reliable inference [4]. Strategies that combine the strengths of a variety of approaches and regularization methods, in situations where the proposed methods may fail, could be used as building blocks in developing more powerful procedures [4].

The proposed approaches were applied to a bladder cancer dataset with gene signature survival data and competing risks. The fitted model based on the subdistribution hazards was shown to identify genes that were related to cancer events. Most of the genes found here have known functions in cancer-related pathways, especially in bladder cancer [59–64]. Although we applied the proposed method over a gene expression data, it can be easily applied to other types of high-dimension data like single-nucleotide polymorphism.

The main objective of the present study was to explore variable selection methods in high-dimensional competing risk data based on the subdistribution hazards. Although, we have focused on the multiplicative hazards model, the techniques here can be adapted to other survival models such as additive hazards approaches, which have promising characteristics. This issue is an interesting topic for future research. In simulations and data analysis of this study, we only considered identical link function from Box-Cox transformation class and one a link function for proportional odds model from logarithmic transformation class, but, comparing and considering other types of link functions (*g*(.)) is another potential topic for future studies which would be interesting with more scenarios. Extension of the proposed model to the cure mixture models is another possible future work.

## Figures and Tables

**Table 1:** Results of the simulation studies for the Fine and Gray model with 5 informative variables (*d* = 5000) for *ρ* = 0.1 scenario. Values shown are means (standard deviations) of each performance measure over 500 replicates (~40% censoring).

*n*	*k* = *I*/*C*	0.2			0.5			0.8		
	Method	No. selected variables	TPR	FPR	No. selected variables	TPR	FPR	No. selected variables	TPR	FPR
200	ALASSO	36.913 (5.119)	0.816 (0.161)	0.007 (0.005)	33.682 (2.255)	0.969 (0.075)	0.006 (0.004)	26.242 (1.796)	0.988 (0.047)	0.004 (0.003)
	AENET	36.532 (4.503)	0.830 (0.174)	0.006 (0.004)	33.571 (1.909)	0.958 (0.089)	0.006 (0.004)	26.636 (1.808)	0.989 (0.046)	0.004 (0.004)
	SCAD	37.684 (4.484)	0.858 (0.162)	0.007 (0.003)	35.414 (2.691)	0.972 (0.073)	0.006 (0.006)	25.054 (2.136)	0.993 (0.042)	0.004 (0.005)
	SCAD-L_2_	37.190 (3.171)	0.867 (0.156)	0.006 (0.003)	35.960 (2.742)	0.960 (0.092)	0.006 (0.006)	25.935 (2.232)	0.994 (0.034)	0.004 (0.005)
	MCP	27.401 (2.996)	0.860 (0.142)	0.004 (0.002)	25.125 (2.308)	0.975 (0.068)	0.004 (0.005)	23.634 (1.889)	0.994 (0.034)	0.004 (0.004)
	MCP-L_2_	27.030 (3.103)	0.849 (0.149)	0.004 (0.003)	25.881 (2.115)	0.971 (0.079)	0.004 (0.004)	23.328 (1.643)	0.995 (0.031)	0.004 (0.004)
	Boosting (Binder)	41.750 (4.874)	0.899 (0.140)	0.007 (0.005)	39.940 (4.890)	0.987 (0.052)	0.007 (0.005)	38.272 (4.731)	1.000 (0.000)	0.007 (0.006)
	Oracle	5.000	1.000	0.000	5.000	1.000	0.000	5.000	1.0000	0.000

400	ALASSO	34.871 (1.430)	0.963 (0.820)	0.006 (0.005)	31.846 (1.196)	1.000 (0.000)	0.005 (0.003)	24.572 (0.891)	1.000 (0.000)	0.004 (0.002)
	AENET	34.181 (1.649)	0.972 (0.075)	0.006 (0.003)	31.701 (1.041)	0.999 (0.014)	0.005 (0.002)	24.631 (0.925)	1.000 (0.000)	0.004 (0.002)
	SCAD	35.192 (1.821)	0.987 (0.053)	0.006 (0.003)	29.942 (1.679)	1.000 (0.000)	0.004 (0.004)	25.304 (1.260)	1.000 (0.000)	0.004 (0.003)
	SCAD-L_2_	35.736 (1.805)	0.980 (0.067)	0.006 (0.005)	29.672 (1.470)	0.998 (0.020)	0.004 (0.003)	25.150 (1.164)	1.00 (0.000)	0.004 (0.003)
	MCP	24.140 (1.580)	0.968 (0.073)	0.004 (0.003)	20.761 (1.227)	0.999 (0.014)	0.003 (0.002)	18.572 (1.228)	1.000 (0.000)	0.003 (0.002)
	MCP-L_2_	24.162 (1.468)	0.972 (0.072)	0.004 (0.003)	20.661 (1.105)	0.998 (0.020)	0.003 (0.002)	18.381 (0.916)	0.999 (0.014)	0.003 (0.002)
	Boosting (Binder)	39.911 (5.256)	0.995 (0.031)	0.007 (0.005)	39.280 (4.874)	1.000 (0.000)	0.007 (0.005)	38.695 (4.965)	1.000 (0.000)	0.007 (0.004)
	Oracle	5.000	1.000	0.000	5.000	1.000	0.000	5.000	1.00	0.00

TPR: true positive rate; FPR: false positive rate; *n*: sample size.

**Table 2:** Results of the simulation studies for the Fine and Gray model with 5 informative variables (*d* = 5000) for *ρ* = 0.5 scenario. Values shown are means (standard deviations) of each performance measure over 500 replicates (*b* = 3: ~40% average censoring).

*n*	*k* = *I*/*C*	0.2			0.5			0.8		
	Method	No. selected variables	TPR	FDR	No. selected variables	TPR	FDR	No. selected variables	TPR	FDR
200	ALASSO	35.702 (1.969)	0.779 (0.136)	0.006 (0.004)	36.054 (2.027)	0.900 (0.116)	0.006 (0.004)	30.261 (2.202)	0.953 (0.085)	0.005 (0.005)
	AENET	35.651 (1.766)	0.775 (0.163)	0.006 (0.003)	35.791 (1.745)	0.891 (0.124)	0.006 (0.004)	30.092 (1.584)	0.937 (0.101)	0.005 (0.003)
	SCAD	36.763 (3.429)	0.740 (0.166)	0.007 (0.006)	36.723 (2.717)	0.908 (0.108)	0.006 (0.006)	27.101 (2.831)	0.940 (0.101)	0.004 (0.003)
	SCAD-L_2_	35.742 (2.458)	0.795 (0.148)	0.006 (0.005)	36.783 (3.299)	0.896 (0.120)	0.006 (0.005)	26.511 (2.062)	0.946 (0.094)	0.004 (0.003)
	MCP	26.234 (4.008)	0.690 (0.139)	0.004 (0.005)	25.870 (1.983)	0.847 (0.124)	0.004 (0.004)	24.384 (1.805)	0.922 (0.102)	0.004 (0.004)
	MCP-L_2_	25.691 (2.124)	0.738 (0.133)	0.004 (0.005)	25.921 (2.146)	0.857 (0.137)	0.004 (0.004)	23.531 (2.654)	0.942 (0.099)	0.004 (0.004)
	Boosting (Binder)	40.524 (4.171)	0.966 (0.076)	0.007 (0.006)	39.447 (3.187)	0.994 (0.034)	0.007 (0.007)	38.602 (4.211)	0.990 (0.044)	0.007 (0.006)
	Oracle	5.000	1.000	0.000	5.000	1.000	0.000	5.000	1.000	0.000

400	ALASSO	37.795 (1.436)	0.942 (0.108)	0.007 (0.003)	35.522 (0.999)	0.990 (0.031)	0.006 (0.002)	29.581 (0.923)	1.000 (0.020)	0.005 (0.002)
	AENET	36.764 (1.534)	0.946 (0.093)	0.006 (0.003)	35.864 (1.137)	0.990 (0.044)	0.006 (0.003)	29.833 (1.234)	1.000 (0.000)	0.005 (0.002)
	SCAD	36.510 (1.956)	0.908 (0.117)	0.006 (0.004)	31.754 (1.774)	0.979 (0.061)	0.005 (0.003)	26.813 (1.523)	0.999 (0.034)	0.004 (0.003)
	SCAD-L_2_	35.722 (1.590)	0.902 (0.113)	0.006 (0.003)	31.122 (1.297)	0.988 (0.048)	0.005 (0.003)	26.181 (1.296)	0.999 (0.034)	0.004 (0.003)
	MCP	25.553 (1.517)	0.874 (0.121)	0.004 (0.003)	22.701 (1.364)	0.963 (0.083)	0.004 (0.003)	20.455 (0.869)	0.992 (0.039)	0.003 (0.002)
	MCP-L_2_	25.382 (1.388)	0.897 (0.115)	0.004 (0.003)	22.571 (0.987)	0.982 (0.057)	0.004 (0.003)	20.482 (1.020)	0.998 (0.020)	0.003 (0.002)
	Boosting (Binder)	39.452 (3.770)	0.994 (0.034)	0.007 (0.008)	37.752 (3.066)	1.000 (0.000)	0.006 (0.006)	38.330 (3.714)	1.00 (0.000)	0.007 (0.006)
	Oracle	5.000	1.000	0.000	5.000	1.000	0.000	5.000	1.000	0.000

TPR: true positive rate; FPR: false positive rate; *n*: sample size.

**Table 3:** Results of the simulation studies for proportional odds model (*g*(*x*) = log(1 + *x*)) with 5 informative variables (*d* = 5000) for *ρ* = 0.1 and *ρ* = 0.5 scenario. Values shown are means (standard deviations) of each performance measure over 500 replicates (*b* = 3: ~40% average censoring; *I*/*C* = 0.5).

	*ρ* = 0.1						No. selected variables	*ρ* = 0.5				FDR
	*n* = 200			*n* = 400				*n* = 200		*n* = 400		
	No. selected variables	TPR	FDR	No. selected variables	TPR	FDR		TPR	FDR	No. selected variables	TPR	
ALASSO	37.233 (3.143)	0.972 (0.073)	0.006 (0.003)	32.445 (2.341)	1.000 (0.000)	0.005 (0.004)	38.113 (2.027)	0.903 (0.087)	0.006 (0.004)	33.271 (2.271)	0.996 (0.033)	0.006 (0.003)
AENET	37.523 (2.881)	0.970 (0.077)	0.006 (0.005)	32.611 (2.254)	1.000 (0.000)	0.005 (0.002)	35.792 (1.745)	0.902 (0.124)	0.006 (0.003)	34.215 (2.421)	0.995 (0.041)	0.006 (0.003)
SCAD	31.231 (2.779)	0.978 (0.067)	0.005 (0.004)	30.472 (2.471)	1.000 (0.000)	0.005 (0.002)	36.723 (2.717)	0.901 (0.108)	0.006 (0.005)	32.344 (2.622)	0.982 (0.031)	0.005 (0.003)
SCAD-L_2_	32.485 (2.812)	0.979 (0.063)	0.005 (0.005)	30.285 (2.345)	0.999 (0.002)	0.005 (0.002)	36.785 (3.299)	0.903 (0.120)	0.006 (0.005)	33.426 (2.312)	0.990 (0.027)	0.006 (0.003)
MCP	27.131 (3.110)	0.980 (0.061)	0.004 (0.005)	24.634 (2.331)	1.000 (0.000)	0.004 (0.002)	25.872 (1.983)	0.907 (0.124)	0.004 (0.004)	25.121 (2.107)	0.990 (0.033)	0.004 (0.003)
MCP-L_2_	27.743 (3.103)	0.982 (0.060)	0.004 (0.005)	24.411 (2.262)	1.000 (0.000)	0.004 (0.002)	25.921 (2.146)	0.912 (0.137)	0.004 (0.004)	25.423 (1.998)	0.996 (0.024)	0.004 (0.003)

**Table 4:** Variable selection results (relative frequency of selection) for different methods for independent variables (~40% censoring) over 500 repetitions.

		*n* = 200							*n* = 400						
*f* = *I*/*C*	Method	*X*_1_	*X*_2_	*X*_3_	*X*_4_	*X*_5_	*X*_6_	FPR^∗^	*X*_1_	*X*_2_	*X*_3_	*X*_4_	*X*_5_	*X*_6_	FPR^∗^
0.5	ALASSO	500	500	0	400	500	0	0.006	500	500	0	450	500	0	0.006
	AENET	500	500	500	500	500	500	0.006	500	500	500	500	500	500	0.006
	SCAD	500	500	0	500	450	0	0.006	500	500	0	500	500	0	0.005
	SCAD-L_2_	500	500	500	500	500	500	0.006	500	500	500	500	500	500	0.005
	MCP	500	500	0	450	500	0	0.004	500	500	0	500	500	0	0.004
	MCP-L_2_	500	500	500	500	500	500	0.004	500	500	500	500	500	500	0.004
	Boosting (Binder)	500	500	0	500	500	0	0.007	500	500	0	500	500	0	0.006

^∗^Average false positive rate (FPR) across all simulations of selection of *βj* = 0, averaged across all *j* ∈ {7, ⋯, 5000}.

**Table 5:** Selected genes data by ENET, AENET, and boosting (from Binder et al.'s study) methods for progression or death from bladder cancer event in bladder cancer data.

Gene ID	GenBank accession no.	Symbol	ALASSO	AENET	SCAD	SCAD-L_2_	MCP	MCP-L_2_	Boosting	Related to cancer
SEQ162	XM_088569	PTGR1		✓		✓		✓	✓	Yes
SEQ164	XM_088569	PTGR1			✓		✓			Yes
SEQ213	NM_004358	CDC25B	✓							Yes
SEQ227	NM_007008	RTN4	✓	✓	✓	✓	✓	✓	✓	Yes
SEQ240	NM_016252	BIRC6		✓	✓					Yes
SEQ248	NM_032333	PRXL2A			✓		✓	✓		Yes
SEQ249	NM_053056	CCND1		✓						Yes
SEQ264	NM_001168	BIRC5			✓					Yes
SEQ265	NM_001168	BIRC5		✓						Yes
SEQ279	XM_027898	PIF1	✓	✓		✓				Yes
SEQ287	AK026169	SLC5A3	✓	✓						Yes
SEQ34	NM_000433	NCF2	✓	✓	✓	✓	✓	✓	✓	Yes
SEQ343	XM_085721	IL6STP1				✓				
SEQ347	NM_001129	AEBP1			✓	✓	✓	✓	✓	Yes
SEQ377	NM_002664	PLEK	✓	✓	✓	✓	✓	✓		Yes
SEQ392	NM_001752	CAT				✓				Yes
SEQ497	NM_004735	LRRFIP1	✓		✓	✓		✓		Yes
SEQ522	M55643	NFKB1				✓				Yes
SEQ634	NM_004453	ETFDH	✓	✓	✓	✓	✓	✓	✓	Yes
SEQ648	NM_006225	PLCD1			✓			✓		Yes
SEQ650	NM_021173	POLD4			✓					Yes
SEQ681	NM_001607	ACAA1		✓			✓		✓	Yes
SEQ709	NM_000089	COL1A2				✓				Yes
SEQ715	AA827892	cDNA clone IMAGE:1367358 3′			✓					
SEQ776	NM_018695.1	ERBIN			✓	✓	✓	✓		
SEQ820	NM_005916	MCM7	✓	✓					✓	Yes
SEQ833	NM_001255.1	CDC20	✓	✓	✓	✓	✓	✓	✓	Yes
SEQ843	NM_000698.1	ALOX5	✓							Yes
SEQ847	NM_018229.2	MUDENG							✓	Yes
SEQ919	NM_024665.2	IRA1			✓		✓	✓		
SEQ921	BE382685.1	cDNA clone IMAGE:3627276 5′			✓	✓	✓	✓		
SEQ940	NM_020159.1	SMARCAD1	✓	✓	✓	✓	✓	✓	✓	Yes
SEQ991	NM_007373.1	SHOC2			✓		✓	✓		
SEQ1028	NM_000228.1	LAMB3			✓					Yes
SEQ1036	NM_012164.2	FBXW2	✓		✓	✓	✓	✓		Yes
SEQ1037	NM_005127.2	CLEC2B	✓	✓		✓				Yes
SEQ1197	NM_003103.5	SON	✓	✓	✓	✓	✓	✓	✓	Yes
SEQ1224	NM_004060.3	CCNG1	✓		✓	✓	✓	✓		Yes
SEQ1226	NM_001921.1	DCTD		✓						Yes
SEQ1262	NM_000875.2	IGF1R	✓	✓	✓		✓	✓	✓	Yes
SEQ1284	NM_002757.2	MAP2K5	✓	✓	✓	✓	✓	✓		Yes
SEQ1325	NM_001085.2	SERPINA3			✓	✓	✓	✓		Yes
No.			18	19	26	22	20	21	12	

**Table 6:** Regression coefficients of six common genes selected by all methods correlated with bladder cancer patients' subdistribution hazards.

Gene symbol	Sequence	Coefficient (SE)	HR^∗^	*P* value
CDC20	SEQ833	0.986 (0.219)	2.680	<0.0001
NCF2	SEQ34	0.905 (0.194)	2.472	<0.0001
SMARCAD1	SEQ940	-0.808 (0.233)	0.446	<0.0001
RTN4	SEQ227	-0.823 (0.322)	0.439	0.011
ETFDH	SEQ634	0.763 (0.321)	2.145	0.018
SON	SEQ1197	0.734 (0.246)	2.083	0.003

^∗^HR: hazards ratio.

## Data Availability

The used data is available from https://www.ncbi.nlm.nih.gov/geo/query/acc.cgi?acc=GSE5479.

## Additional Points

*Key Points*. (i) Analysis of high-dimensional competing risks requires models that consider the event of interest and the competing events simultaneously, while also dealing with censoring. (ii) A likelihood-based penalized approach is extended for direct variable selection under the subdistribution hazards model for high-dimensional competing risk data. (iii) Some widely used penalties, including ALASSO, AENET, SCAD, and MCP, and their L_2_ counterparts called SCAD-L_2_ and MCP-L_2_ were considered. (iv) Simulation studies showed that the proposed methods performed effective in identifying important variables in high-dimension competing risk data. (v) Analysis of a real genomic competing risk dataset obtained from patients with bladder cancer revealed a set of genes associated with the incidence of death.

## Supplementary Materials

Supplementary Materials Table S1: simulation results for different choices of *β*_1_ and *β*_2_ for the Fine and Gray model (five informative variables; *d* = 5000, *ρ* = 0.5; *n* = 400; censoring rate at 40%; *K* = *I*/*C* = 0.5). Values shown are means (standard deviations) of each performance measure over 500 replicates.
