# Supplementary material for: Regularized Weighted Nonparametric Likelihood Approach for High-Dimension Sparse Subdistribution Hazards Model for Competing Risk Data
Source: Comput Math Methods Med. 2021 Sep 19;2021:5169052. doi: 10.1155/2021/5169052 (PMC8476266; doi:10.1155/2021/5169052)
Supplement: Supplementary Materials — Table S1: simulation results for different choices of β1 and β2 for the Fine and Gray model (five informative variables; d = 5000, ρ = 0.5; n = 400; censoring rate at 40%; K = I/C = 0.5). Values shown are means (standard deviations) of each performance measure over 500 replicates. [file 5169052.f1.docx]

Table S1**:** Simulation results for different choices of $\beta_{1}$ and $\beta_{2}$for Fine and Gray model (five informative variables; d = 5000$\rho=0.5$; n=400; censoring rate at 40%; K=I/C=0.5). Values shown are means (standard deviations) of each performance measure over 500 replicates.

| k= I/C | 0.2 | | | 0.5 | | | 0.8 | | |
| --- | --- | --- | --- | --- | --- | --- | --- | --- | --- |
| Method | No. Selected variables | TPR | FPR | No. Selected variables | TPR | FPR | No. Selected variables | TPR | FPR |
| ALASSO | 34.41  (1.422) | 0.963  (0.821) | 0.006  (0.005) | 31.86  (1.198) | 1.00  (0.000) | 0.005  (0.003) | 24.58  (0.893) | 1.00  (0.000) | 0.004  (0.002) |
| AENET | 34.13  (1.651) | 0.972  (0.074) | 0.006  (0.003) | 31.71  (1.040) | 0.999  (0.013) | 0.005  (0.002) | 24.64  (0.928) | 1.00  (0.000) | 0.004  (0.002) |
| SCAD | 35.20  (1.822) | 0.987  (0.052) | 0.006  (0.003) | 29.93  (1.680) | 1.00  (0.000) | 0.004  (0.004) | 25.33  (1.258) | 1.000  (0.000) | 0.004  (0.003) |
| SCAD-L_2_ | 35.71  (1.803) | 0.980  (0.069) | 0.006  (0.005) | 29.68  (1.469) | 0.998  (0.022) | 0.004  (0.003) | 25.14  (1.163) | 1.00  (0.000) | 0.004  (0.003) |
| MCP | 24.13  (1.577) | 0.968  (0.073) | 0.004  (0.003) | 20.74  (1.229) | 0.999  (0.016) | 0.003  (0.002) | 18.59  (1.229) | 1.000  (0.000) | 0.003  (0.002) |
| MCP-L_2_ | 24.17  (1.473) | 0.972  (0.073) | 0.004  (0.003) | 20.67  (1.108) | 0.998  (0.024) | 0.003  (0.002) | 18.36  (0.914) | 0.999  (0.013) | 0.003  (0.002) |
| Boosting (Binder) | 39.90  (5.252) | 0.995  (0.032) | 0.007  (0.005) | 39.29  (4.880) | 1.00  (0.000) | 0.007  (0.005) | 38.71  (4.961) | 1.00  (0.000) | 0.007  (0.004) |
| Oracle | 5 | 1.00 | 0.00 | 5 | 1.00 | 0.00 | 5 | 1.00 | 0.00 |

TPR: True positive rate; FPR: False positive rate; n=sample size; I/C: Interested event/competing event
